# Extra-gastrointestinal stromal tumors (EGISTs): A case report for a mischief entity

**DOI:** 10.1097/MD.0000000000033394

**Published:** 2023-03-31

**Authors:** Mohamad Hadi El Charif, Sara Amro, Fouad Boulos, Mohamad Khalife, Ali Shamseddine, Hazem Assi, Eman Sbaity

**Affiliations:** a Department of Internal Medicine, American University of Beirut Medical Center, Beirut, Lebanon; b Department of Surgery, American University of Beirut Medical Center, Beirut, Lebanon; c Department of Pathology, American University of Beirut Medical Center, Beirut, Lebanon.

**Keywords:** case report, EGIST, extra-gastrointestinal stromal tumor, histology, soft tissue sarcoma, subtype

## Abstract

Extra-gastrointestinal stromal tumor is a rare subtype of soft tissue sarcomas with significantly variable presentation, management, and prognosis. This makes it crucial to report the different institutional experiences of encountering extra-gastrointestinal stromal tumors (EGIST).

**Case presentation:**

We report 3 cases of EGIST diagnosis at American University of Beirut Medical Center for 2 males and 1 female in the 5th, 6th, and 7th decades of life, respectively. For the first case, the tumor was initially suspected to be ovarian cancer, but biopsy revealed a diagnosis of EGIST, and the patient was started on neoadjuvant therapy. In the second case, the tumor was retro-gastric and prelim diagnosis was gastric cancer but again biopsy revealed an EGIST histopathology, and the patient underwent surgery and adjuvant treatment. For the third case, a previous history of testicular cancer prompted an initial suspicion of recurrence with metastasis but biopsy and immunohistochemistry staining revealed EGIST with related markers. The patient underwent treatment at a different institution in his home country.

**Conclusion:**

This report sheds light on the importance of keeping EGIST amongst any differential list for abdominal and pelvic tumors. It also shows that EGIST-focused studies are needed to assess the effectiveness of the different treatment modalities available when utilized specifically for EGIST. This would allow for better oncological outcomes and improved quality of life.

## 1. Introduction

Gastrointestinal stromal tumors (GIST) are a unique subtype of subepithelial soft tissue neoplasms of the gastrointestinal tract (GIT). They are the most common mesenchymal tumors of the GIT, occurring most frequently in the stomach and small intestine.^[1]^ Extra-gastrointestinal stromal tumors (EGIST) are an extremely rare entity along the GIST spectrum, accounting for only 5% to 10% of all GISTs and occurring most commonly in the omentum or mesentery.^[2]^ This rare subtype of GIST is reported in only few case reports of the literature.

We report 3 cases in which EGIST occurred in the patients’ omentum or peritoneal cavity and presented at the American University of Beirut Medical Center.

## 2. Methods

This study was granted approval by the Institutional Review Board (IRB) at American University of Beirut Medical Center. The requirement for informed consent was waived based on the premise that data on all patients was de-identified and collected via retrospective medical chart review and we did not contact patients for any research purposes.

## 3. Case 1

**Figure 1.**
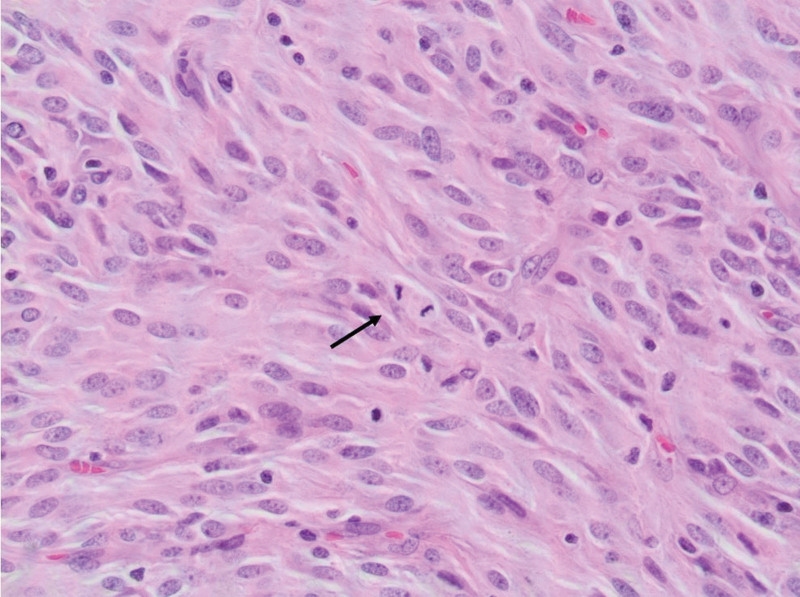
Ovarian EGIST: H&E stain of GIST showing spindle cells with small nucleoli and abundant fibrillary amphophilic cytoplasm. Mitotic activity is evident (arrow). EGIST = extra-gastrointestinal stromal tumors, GIST = gastrointestinal stromal tumors, H&E = hematoxylin and eosin.

**Figure 2.**
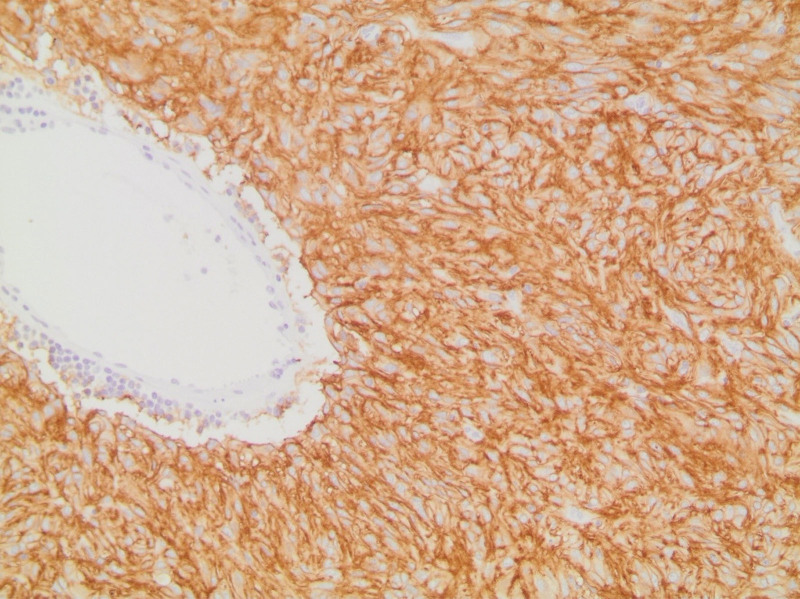
Ovarian EGIST: Diffuse immunohistochemical staining for DOG-1 in ovarian GIST. DOG-1 = discovered on GIST 1, EGIST = extra-gastrointestinal stromal tumors, GIST = gastrointestinal stromal tumors.

The first case is a 78-year-old female who presented with abdominal bloating, heaviness, and nausea. The patient medical history is significant for gastroesophageal reflux disease but no personal malignancy history. Physical examination was nonrevealing. Imaging work-up revealed bilateral adnexal masses with omental metastasis. However, upon laparoscopic excisional biopsy of a peritoneal lesion, pathology revealed a tumor with spindle/polygonal cell morphology (Fig. 1), cluster of differentiation (CD117) and discovered on GIST 1 (DOG-1) positivity (Fig. 2) and negative CD34 immunohistochemistry, consistent with a diagnosis of extra-gastrointestinal stromal tumor of the omentum metastatic to the ovaries and bilateral iliac lymph nodes. The patient was started on neoadjuvant therapy with Imatinib. Follow-up CT scan done 2 years following treatment revealed active and progressive disease. The patient was subsequently lost to follow-up.

## 4. Case 2

**Figure 3.**
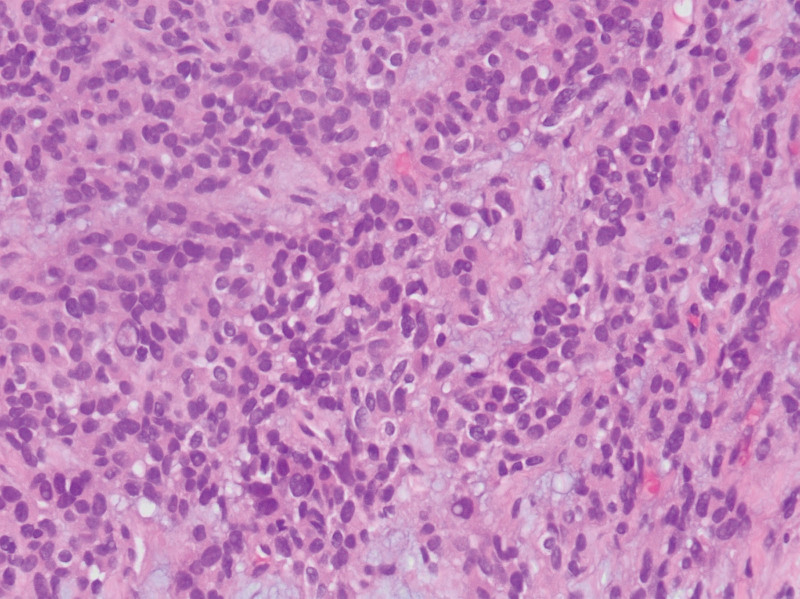
Retro-gastric EGIST: H&E stain showing epithelioid to spindle atypical cells with variably vacuolated cytoplasm. EGIST = extra-gastrointestinal stromal tumors, H&E = hematoxylin and eosin.

**Figure 4.**
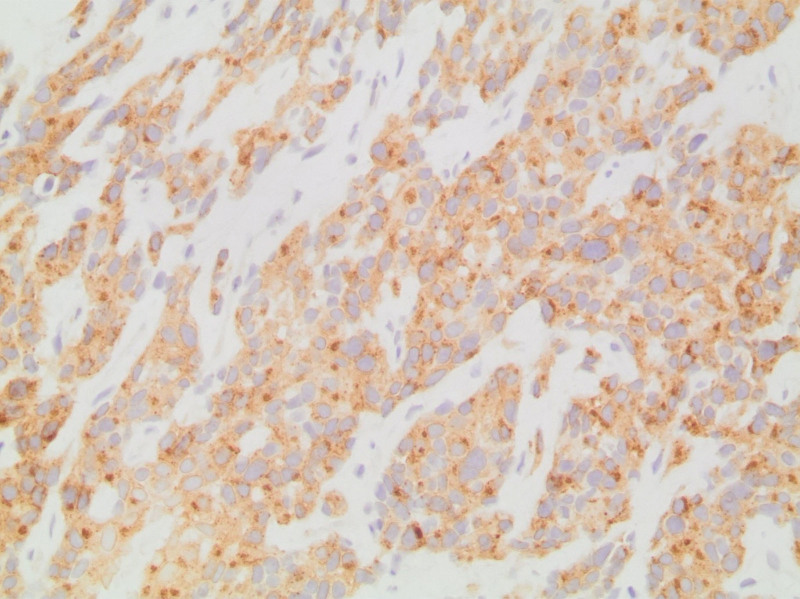
Retro-gastric EGIST: CD117 (c-kit) immunohistochemical stain showing moderate positivity in the tumor cells with a diffuse and dot-like cytoplasmic pattern. CD = cluster of differentiation, EGIST = extra-gastrointestinal stromal tumors.

The second case is a 57-year-old male who presented with bloating, diarrhea, abdominal distention, and significant unintentional weight loss of 20 kg. Past medical history is significant for chronic kidney disease. Family history is significant for gastric and uterine cancers. Physical exam revealed a diffusely tender abdomen, mainly in the epigastric area. CT scan of the abdomen and pelvis revealed a retro gastric mass with peritoneal seeding and metastasis to the liver. Biopsy showed epithelioid undifferentiated cells (Fig. 3) that were CD117 positive (Fig. 4) and CD34 negative. This led to the diagnosis of EGIST. Exploratory laparotomy was done and a 4 × 3.9 cm mass was resected through subtotal gastrectomy and retro-gastric mass removal. Patient received Imatinib adjuvant therapy for 1 year. Follow-up PET scan revealed disease progression, and imatinib therapy was resumed. Unfortunately, the patient passed away from his disease 6 months later.

## 5. Case 3

**Figure 5.**
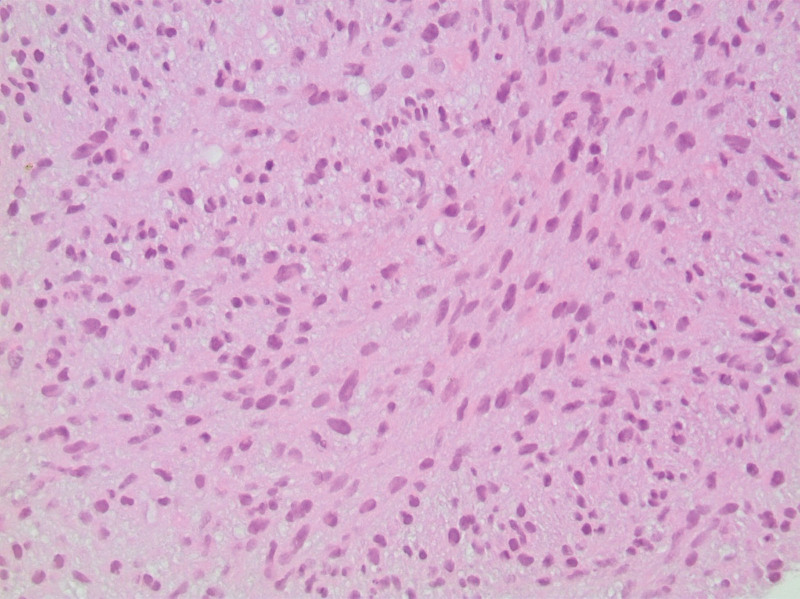
Peritoneal EGIST: GIST tissue showing atypical spindle cells on H&E stain with abundant eosinophilic cytoplasm and elongated nuclei. EGIST = extra-gastrointestinal stromal tumors, GIST = gastrointestinal stromal tumors, H&E = hematoxylin and eosin.

**Figure 6.**
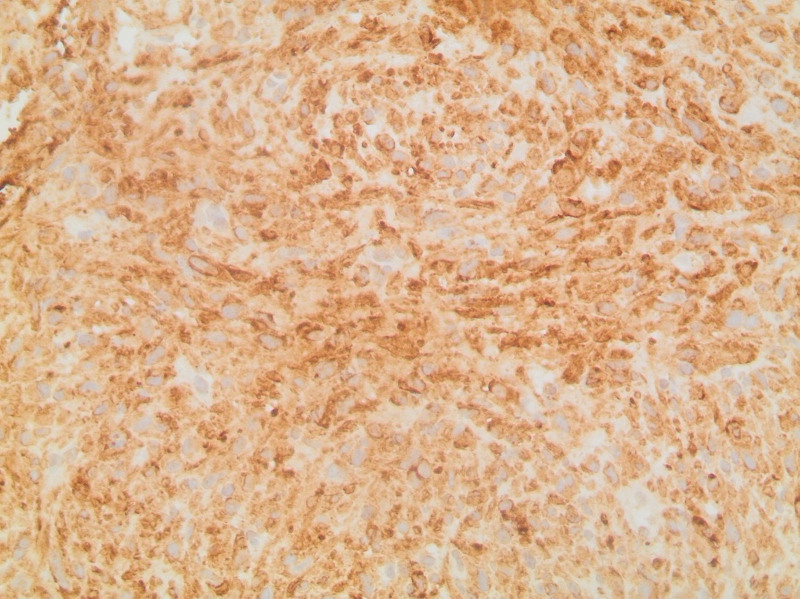
Peritoneal EGIST: DOG-1; immunohistochemical stain showing strong and diffuse cytoplasmic positivity. EGIST = extra-gastrointestinal stromal tumors, DOG-1 = discovered on GIST 1.

The third case we report herein is a 66-year-old Iraqi male who presented with decreased appetite and constipation. Past medical history is significant for hypertension, coronary artery disease, and testicular cancer. PET scan revealed a 9 × 5.4 cm peritoneal mass with metastasis to the liver, bone, and lungs. CT-guided biopsy revealed spindle cells (Fig. 5) with immunohistochemistry staining positive for DOG-1 (gene encoding chloride channel protein Anoctamin 1) (Fig. 6) and CD117, and negative for CD34, thus the mass is diagnosed as EGIST. The patient elected to pursue treatment in his home country.

## 6. Discussion

EGIST is a rare type of GIST with recent studies revealing that it is even rarer than thought before (<1.5%). Several case reports are identified in the literature from around the world describing encounters with EGIST. Anatomically, cases seem to maintain a relatively close geographic relationship to the GIT, most commonly occurring in the omentum or mesentery and to a lesser degree in the retroperitoneum.^[3]^ This is similar to the locations we described for our 2 cases. Just like GISTs, EGISTs arise from the interstitial cells of Cajal, which can occur ectopically in different intraabdominal locations.^[4,5]^ Another theory about the origin of EGIST is described by Agaimy et al, who reported that it is necessary to look for any adhesions or focal attachments between the stromal tumor and the GI tube during surgery as they theorize that EGIST is a result of excessive intramural growth that leads to detachment from the GI muscular cover.^[6]^

All our cases presented in a similar manner, with symptoms related to a GIT pathology including abdominal discomfort, bloating and constipation. EGISTs rarely manifest symptoms when small, and only when they are large enough (>10 cm) or metastatic do they start causing symptoms such as GIT obstruction, nausea, or abdominal discomfort.^[2,7,8]^ Although there are no specific risk factors for EGISTs, it has been reported that similar to GISTs, age, gender and genetic mutations play a role in EGIST pathogenesis.^[1,2]^ Our cases conform to this with all 3 of our patients presenting at an age above 50 years and male predominance (2:1). While 2 out of 3 patients had a CD117 (c-kit gene mutation) positive on immunohistochemical testing, the literature reports that more than 95% of cases carry a c-kit mutation which plays a pivotal role in defining GIST.^[9]^ On the other hand, CD34 was negative in all our patients, although some studies have shown that CD34 is positive in 70% of EGIST cases.^[10]^ The suspicion of EGIST was present on imaging of 2 cases from our series but the confirmation of its presence, in all the cases, was only possible after surgical pathology completion. Thorough radiological assessment is pivotal in the diagnosis of abdominal neoplasms, but it may still be difficult to differentiate a neoplastic occurrence in the lesser omentum from an occurrence in the gastric lesser curvature thus delaying the final diagnosis until surgery.^[11]^ In addition, few papers have reported instances where a radiologically suspected ovarian tumor turned out to be an EGIST “in-disguise.”^[12]^ The differential diagnosis for a peritoneal mass on imaging includes carcinoma, melanoma, lymphoma, neuroendocrine tumor, mesothelioma, perivascular epithelioid cell tumor and others. As such, the gold standard for a diagnosis of EGIST is the same as its GIT counterpart namely via biopsy and immunohistochemical studies. Finding of spindle, epithelioid or mixed cells in an omental or peritoneal mass biopsy raises the consideration of EGIST. This suspicion of EGIST is further confirmed with positive staining for CD117 and/or DOG-1.^[2]^ Laboratory studies such as blood workup and serum tumor markers are seldom useful in the diagnosis of GISTs or EGISTs unless done to rule out other types of abdominal neoplasms such as neuroendocrine tumors, teratomas, and others.^[13]^ The management of EGIST in our series encompassed both medical and surgical modalities of treatment. These included neoadjuvant and adjuvant therapy with immuno-targeting molecules as well as radical resection of the tumor. Treatment of EGISTs that are diagnosed early is radical resection of the tumor, which is the only known curative treatment. for, On the other hand, targeted therapy with molecules such as imatinib or sunitinib is reserved for advanced cases and proves most effective in those with C-kit positive mutations.^[3,11,14]^ The use of tyrosine kinase inhibitors has shown to prolong the median survival to 57 months^[12]^ however, EGISTs have been shown to have a significantly greater malignant potential and worse prognosis compared to GISTs. Despite treatment with surgery and systematic therapy with imatinib ±sunitinib, 2 of our patients showed poor response to the treatment with active and progressive disease at a median follow-up time of 21 months. The latter was the case for one of our patients who would be classified in the low-risk group with a tumor size well below 10 cm and that is not associated with necrosis or ulceration while the other belongs to the high-risk group with a tumor size of 9 cm. Having said that, EGIST is better classified as low versus high-risk malignancies depending, in part, on tumor size and histology. Larger tumors with diameters >10 cm, and necrotic or ulcerative tumors define the high-risk group.^[15,16]^

## 7. Conclusion

In this case series we added new data on an uncommonly described nor reported clinical diagnosis of EGIST. This will help fill some of the knowledge gap on the topic and provide investigators, surgeon, and gastroenterologist with valuable data on presentation, diagnosis, treatment, and prognosis of this rare entity.

## Acknowledgments

The author Eman Sbaity would like to acknowledge the training received under the Scholars in HeAlth Research Program (SHARP) that set the required foundations for a career in clinical and translational research. Research reported in this publication was supported by the Fogarty International Center and Office of Dietary Supplements of the National Institutes of Health under Award Number D43 TW009118. The content is solely the responsibility of the authors and does not necessarily represent the official views of the National Institutes of Health.

## Author contributions

**Conceptualization:** Mohamad Hadi El Charif.

**Data curation:** Mohamad Hadi El Charif, Sara Amro, Mohamad Khalife, Ali Shamseddine, Hazem Assi.

**Supervision:** Eman Sbaity.

**Writing – original draft:** Mohamad Hadi El Charif, Sara Amro, Fouad Boulos.

**Writing – review & editing:** Mohamad Hadi El Charif.
